# Genome-wide association study suggests impact of chromosome 10 rs139401390 on kidney function in patients with coronary artery disease

**DOI:** 10.1038/s41598-019-39055-y

**Published:** 2019-02-26

**Authors:** Boris Schmitz, Marcus E. Kleber, Malte Lenders, Graciela E. Delgado, Christiane Engelbertz, Jie Huang, Hermann Pavenstädt, Günter Breithardt, Stefan-Martin Brand, Winfried März, Eva Brand

**Affiliations:** 1 Institute of Sports Medicine, Molecular Genetics of Cardiovascular Disease, University Hospital Muenster, Muenster, Germany; 2 Medical Clinic V, Mannheim Medical Faculty, University of Heidelberg, Mannheim, Germany; 3 Institute of Nutrition, Friedrich Schiller University Jena, Jena, Germany; 4 Internal Medicine D, Department of Nephrology, Hypertension and Rheumatology, University Hospital Muenster, Muenster, Germany; 5 Division of Vascular Medicine, Department of Cardiovascular Medicine, University Hospital Muenster, Muenster, Germany; 6 Department of Human Genetics, Wellcome Trust Sanger Institute, Hinxton, Cambridge UK; 7 Clinical Institute of Medical and Chemical Laboratory Diagnostics, Medical University of Graz, Graz, Austria; 8 Synlab Academy, Synlab Holding Deutschland GmbH, Mannheim, Germany

## Abstract

Chronic kidney disease (CKD) is an independent risk factor for onset and progression of coronary artery disease (CAD). Discovery of predisposing loci for kidney function in CAD patients was performed using a genome-wide association approach. Inclusion criteria were CAD with ≥50% stenosis (≥1 coronary artery) and a creatinine-based estimated glomerular filtration rate (eGFR) of 30–75 ml/min/1.73 m^2^. An association of rs139401390 located to a region 58.8 kb upstream of *renalase* (*RNLS*) with eGFR was detected in the Ludwigshafen Risk and Cardiovascular Health (LURIC) study (n = 499, p = 7.88 × 10^−9^, mean eGFR 60.7 ml/min/1.73 m^2^). Direct genotyping of rs139401390A > G suggested increased eGFR by 12.0 ml/min/1.73 m^2^ per A allele (p = 0.000004). Genome-wide replication of rs139401390A > G in the Coronary Artery Disease and Renal Failure (CAD-REF) registry with a mean eGFR of 47.8 ml/min/1.73 m^2^ (n = 574, p = 0.033) was only nominally significant. Comparison of rs139401390 genotypes for risk of reduced kidney function in the overall LURIC study revealed higher adjusted odds ratios (OR) for eGFR <60 ml/min/1.73 m^2^ for CAD patients (n = 1992, OR = 2.36, p = 0.008, G/A + G/G vs A/A) compared to patients with/without CAD (n = 2908, OR = 1.97, p = 0.014, G/A + G/G vs A/A). No significant risk elevation was detected in patients without CAD (n = 948, p = 0.571). rs139401390 may affect kidney function in CAD patients with mild reduction in eGFR.

## Introduction

Chronic kidney disease (CKD) is a worldwide emerging health problem which affects more than 1 out of 10 adults in the general population of developed countries^1–3^. Most recently, CKD has been established as a new non-traditional and independent risk factor for onset and progression of cardiovascular disease (CVD)^4–6^. Impaired kidney function can be diagnosed by the estimation of glomerular filtration rate (eGFR) and the risk of CVD events increases with decreasing GFR^7,8^. This observation translates into clinical consequences as most CKD patients die of CVD before reaching end-stage renal disease^9^. While the deleterious impact of end-stage renal disease and, more recently, the harmful effects of CKD on CVD have been reported, the clinical course of mild-to-moderate renal impairment remains poorly understood^10^ and interdependent pathophysiological mechanisms of CKD and CVD are largely unknown.

The identification of candidate genes involved in impaired kidney function in the presence of CVD could offer insight into the pathogenesis of this reno-cardiac disease entity and would reveal potential therapeutic targets. However, genome-wide association studies (GWAS) of renal function traits have so far been performed with a focus on the general population^11,12^. Moreover, a recent bidirectional cross-trait single nucleotide polymorphism (SNP) analysis^13^ testing the hypothesis that genetic variants replicatively associated with renal function might increase the risk of vascular disease and *vice versa* reported minimal overlap of risk variants. Out of 19 analyzed SNPs associated with kidney function and 64 validated vascular SNPs, only one vascular locus (*SH2B3*) was significantly associated with eGFR while no association of kidney variants with vascular traits was observed^13^.

Thus, we examined genetic associations in a selected group of patients with a comorbidity of coronary artery disease (CAD) and impaired kidney function.

## Methods

### Patient classification and study design

The study was designed as a cross-sectional analysis of eGFR as continuous trait in patients with CAD recruited in a clinical setting. Inclusion criteria for the primary analysis were a creatinine-based mild impairment of kidney function with an eGFR of 30–75 ml/min/1.73 m^2^ and angiographically documented ≥50% stenosis of at least one coronary artery. The discovery study sample derived from 3,316 participants of the Ludwigshafen Risk and Cardiovascular Health Study (LURIC) whose characteristics have been described in detail elsewhere^14^. In brief, LURIC is a German cohort study designed to investigate biochemical and genetic cardiovascular risk factors. Patients referred to coronary angiography had been consecutively recruited at the Ludwigshafen Heart Center between July 1997 and January 2000. The LURIC study was approved by the ‘Landesärztekammer’ Ethics Committee of Rheinland-Pfalz, Germany (reference 837.255.97). All patients gave written informed consent for participation. For the current analysis, 499 CAD patients with a mild reduction in kidney function were selected for a genome-wide association study (GWAS) on eGFR (discovery cohort). The mean creatinine-based eGFR of the LURIC discovery study sample was 60.7 ml/min/1.73 m^2^.

The independent replication study sample derived from 3,352 patients of the Coronary Artery Disease and Renal Failure registry (CAD-REF) whose characteristics have been described in detail elsewhere^10,15^. The CAD-REF registry is a prospective observational German multicenter study. Patients had been enrolled at 32 qualified German centers between January 2008 and May 2011, coordinated at the University Hospital of Muenster. All investigations in CAD-REF were performed after approval of local ethics committee of the medical association Westfalen-Lippe and the Westphalian Wilhelms-University of Muenster, Germany (reference 2007-315-f-S). Genome-wide data was available for 574 randomly selected CAD-REF study participants of CKD stage 3 (creatinine-based eGFR 30–59 ml/min/1.73 m^2^). The mean creatinine-based eGFR of the CAD-REF study sample was 47.8 ml/min/1.73 m^2^. All experiments were performed in accordance with relevant guidelines and regulations.

### Clinical assessment

Both studies enrolled subjects of European ancestry and determined patients’ eGFR using the creatinine-based “Modification of Diet in Renal Disease” (MDRD) equation. Creatinine was determined from a single serum measurement at the first clinical visit. In LURIC, creatinine was determined by liquid chromatography/mass spectrometry (LC/MS). In CAD-REF, creatinine was determined using the enzymatic peroxidase-antiperoxidase (PAP) method. CAD was defined as angiographically documented >50% stenosis of at least one coronary artery at the first clinical visit. Essential hypertension was defined as systolic blood pressure >140 mm Hg, diastolic blood pressure >90 mm Hg, or the use of antihypertensive drugs. Hyperlipidemia was defined as a total plasma cholesterol level >200 mg/dl, or use of lipid-lowering drugs. Diabetes mellitus was defined as increased fasting (≥126 mg/dl) and/or post-challenge (2 h after the 75 g glucose load > 200 mg/dl) glucose and/or elevated glycated haemoglobin (>6.5%) and/ or history of diabetes/ use of antidiabetic drugs^14,15^.

### Genomic data and genotyping

In both cohorts, genomic DNA was prepared from patients’ peripheral blood. LURIC data were generated using the Affymetrix Human SNP Array 6.0. For the CAD-REF study, the Illumina Omni 2.5-Quad Chip was used. SNPs were excluded in case of a low genotyping call rate (<0.95), Hardy-Weinberg-Equilibrium p < 10^−6^ and minor allele frequency < 0.01. PLINK was used to test samples for relatedness. In the case of a PI-HAT > 0.3 the sample with the lower call rate was excluded. Both datasets were imputed to the 1000 G EUR reference panel (March 2012, v3) using MACH^16^. The analysis included >10 million SNPs and small InDels of high quality (r^2^ > 0.3). Chromosomal positions are based on GRCh37.3. For further statistical analyses using SPSS, the best-guess genotypes for rs139401390 were imported into the database containing the clinical data. Best-guess genotypes could only be determined with high confidence for 489 samples. Thus, for the remaining 10 samples, rs139401390 genotypes were set to ‘missing’. Direct genotyping of the lead SNP rs139401390 was performed using TaqMan SNP genotyping assay on a real-time PCR System ABI7900 (Life Technologies Corporation, Carlsbad, USA) in a 384 well format according to manufactures instructions.

### Statistical analysis

Hardy-Weinberg equilibrium was examined using chi-square test. Categorical data are presented as *n* (percent) of subjects in each group. Continuous data are presented as means ± SD. SPSS version 19.0 (IBM Corporation, Armonk, USA) statistical software package was used. The genome-wide analysis was performed using the software ProbABEL^17^ with the additive genetic model and adjustment with adjustments for age and sex. Adjustment for the first three principle components was used to adjust for population substructure. The genomic inflation factor lambda in LURIC was 1.037. No transformation for eGFR distribution was performed. QQ and Manhattan plots were drawn for the analysis of the results using the R-package “qqman”. Regional plots were drawn using Locuszoom^18^. The P value for genome-wide significance was set to p < 5 × 10^−8^, which corresponds to an α of 0.05 with a Bonferroni correction for one million tests. Suggestive significant SNPs with a p value < 1 × 10^−6^ in the discovery analysis were selected for replication. SNPs with a P value < 0.0055 (Bonferroni correction for nine SNPs) in the replication cohort were regarded to mark suggestive significant loci.

## Results

**Figure 1:**
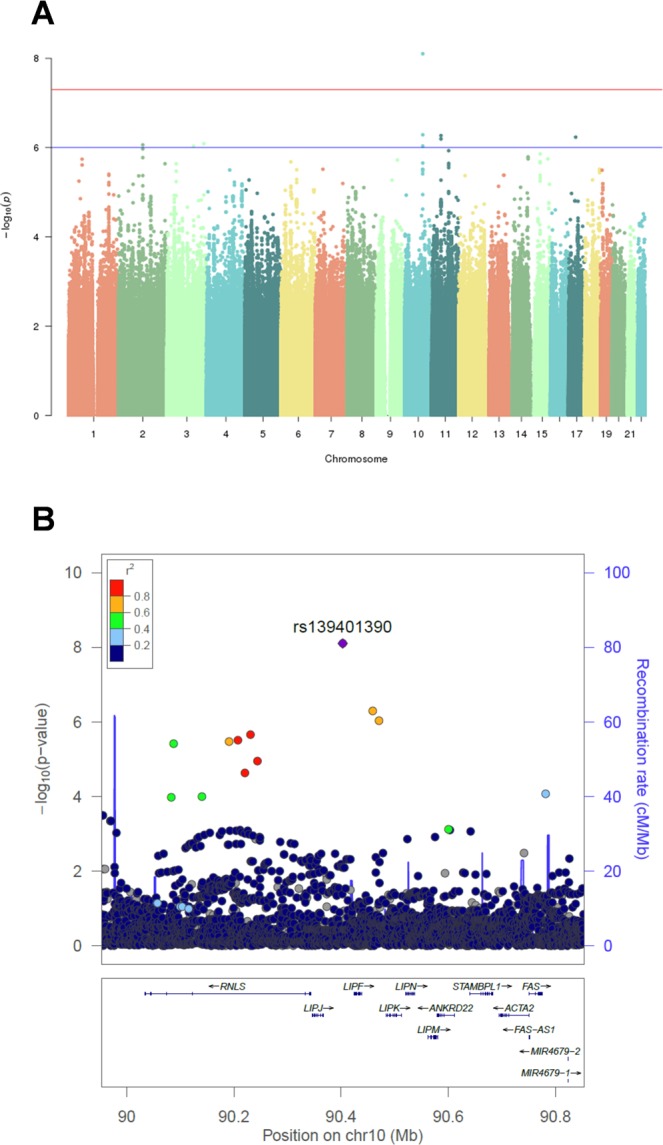
Results of the genome-wide association analysis in the LURIC discovery sample. (**A**) Manhattan plot of the genome-wide association scan. P values corrected for age and sex are shown for each tested SNP. For each chromosome, the results are plotted left to right. The preset threshold for genome-wide significance (P < 5 × 10^−8^) is indicated by a red line. The threshold for suggestive association without genome-wide significance (P < 1 × 10^−6^) is indicated by a blue line (**B**) Association map (prepared using Locuszoom), generated from genotyped and imputed SNPs, centered at rs139401390. SNPs in red are at r^2^ ≥ 0.8 with rs139401390; SNPs in green are at r^2^ = 0.4 – 0.6 and SNPs in light blue are at r^2^ = 0.2–0.4 with the leading SNP. Genes in the region are marked below.

**Figure 2:**
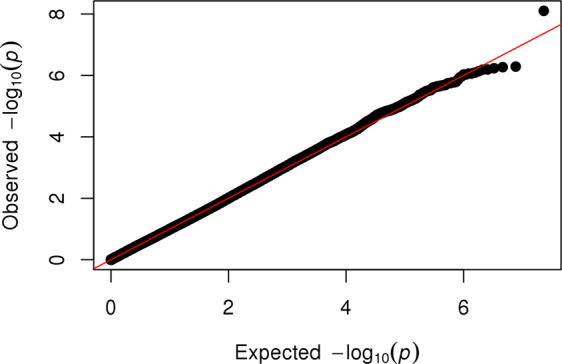
Quantile-quantile (QQ) plot of observed vs. expected ordered −log_10_ (P values) from the discovery analysis. Lower P values correspond to higher −log_10_ (P values). Black: results from discovery analysis; red: null hypothesis.

The condensed clinical characteristics of the LURIC discovery sample and the independent CAD-REF registry replication sample are summarized in Table 1. Patients included in the primary analysis suffered from mildly impaired kidney function and CAD (≥50% stenosis in at least one coronary artery). The mean creatinine-based eGFR in the discovery study sample (LURIC = 499) was 60.7 ml/min/1.73 m^2^ and in the replication study sample (CAD-REF = 574) 47.8 ml/min/1.73 m^2^ (Table 1). The SNP most strongly associated with eGFR was detected on chromosome 10 (lead SNP rs139401390A > G, p = 7.88 × 10^−9^; Figs 1 and 2, Table 2). This variant is located to an intergenic region 58.8 kb upstream of the renal enzyme renalase coding region (*RNLS*). Additional suggestive regions of association were located to chromosomes 2q14.2 (rs138730015G, p = 8.68 × 10^−7^, *Gli2*), 11p11.2 (rs10838518T, p = 5.38 × 10^−7^, *SLC35C1/CRY2*), 3q22.1 (rs202202968A, p = 9.27 × 10^−7^, *ACCP*) and 3q27.1 (rs77080042C, p = 8.14 × 10^−7^, *YEATS2*).

**Table 1:** Study characteristics by study sample.

	LURIC study (n = 499)	CAD-REF registry (n = 574)	P-value*
Age, yrs	68.3 ± 8.70	73.2 ± 7.7	<0.001
Female (%)	32.7	32.4	0.948
eGFR, ml/min/1.73 m^2^	60.7 ± 11.0	47.8 ± 8.46	<0.001
Systolic BP, mmHg	145 ± 25.2	133 ± 21.6	<0.001
Diastolic BP, mmHg	80.5 ± 11.8	75.8 ± 11.5	<0.001
Hypertension (%)	82.6	98.3	<0.001
Total cholesterol, mg/dl	188 ± 40.1	172 ± 43.9	<0.001
Hyperlipidemia (%)	73.5	76.6	0.153
Diabetes (%)	50.1	43.8	0.022
Current smokers (%)	16.0	18.1	0.366

^*^T-test for continuous variables, χ^2^-test for categorical variables. BP: blood pressure; CAD-REF, Coronary Artery Disease and Renal Failure registry (eGFR inclusion, 30–59 ml/min/1.73 m^2^); eGFR: estimated glomerular filtration rate; LURIC, Ludwigshafen Risk and Cardiovascular Health study (eGFR inclusion, 30–75 ml/min/1.73 m^2^).

**Table 2:** Associations with eGFR in stage 1 discovery and stage 2 replication.

SNP ID	Risk allele	Locus genes	Chromosome location	LURIC						CAD-REF						Combined
				Effect allele frequency	n	beta^*^	SE	P	r^2^	Effect allele frequency	n	beta^*^	SE	P	r^2^	P
rs138730015	G	*GLI2*	2:121515159	0.011	499	29.601	6.017	8.68E-07	0.50	0.012	574	6.130	3.088	0.047	0.55	2.15E-06
rs202202968	A	*ACPP*	3:132061495	0.038	499	11.566	2.357	9.27E-07	—	—	—	—	—	—	—	—
rs77080042	C	*YEATS2*	3:183469160	0.092	499	7.292	1.479	8.14E-07	0.65	0.085	574	−0.273	1.001	0.785	0.78	0.001786
rs139401390	G	*RNLS; LIPL1; LIPF*	10:90403139	0.027	499	−16.085	2.787	**7.88E-09**	0.65	0.025	574	−3.720	1.745	0.033	0.82	6.05E-08
rs144118602	T	*RNLS; LIPL1; LIPF*	10:90459083	0.016	499	−17.289	3.444	5.15E-07	0.77	0.017	574	−3.332	2.117	0.116	0.84	0.000006
rs140842984	T	*RNLS; LIPL1; LIPF*	10:90470727	0.015	499	−16.630	3.389	9.24E-07	1.00	0.017	574	−3.420	2.134	0.109	0.84	0.000008
rs10838518	T	*SLC35C1; CRY2*	11:45834466	0.017	499	−13.577	2.709	5.38E-07	0.92	0.019	574	0.455	−1.825	0.803	1.00	0.000381
rs73466394	G	*SLC35C1; CRY2*	11:45838255	0.016	499	−13.306	2.672	6.39E-07	0.98	0.019	574	0.456	−1.825	0.803	1.00	0.000414
rs1662780	T	gene desert	17:35159911	0.419	499	4.641	0.929	5.82E-07	0.63	0.454	574	0.001	0.610	0.999	0.71	0.000766

*ml/min/1.73 m^2^. Base pair position according to the 1000 G EUR reference panel (March 2012, v3). Significant P-values shown in bold.

Since the identification of rs139401390 was based on genotype imputation, direct genotyping of the lead SNP was performed in the discovery sample (n = 499). The correlation between imputed genotype and genotype determined by direct genotyping was 99.2%. A subsequent recalculation suggested an effect of rs139401390 on eGFR by +12.0 ml/min/1.73 m^2^ eGFR per major A (p = 0.000004). SNP rs139401390 was selected for independent replication in the CAD-REF study. The replication analysis suggested that rs139401390 was significantly associated with eGRF (p = 0.033; Table 2). However, the association was not significant after Bonferroni correction (p > 0.0055) and correlation between imputed genotype and genotype determined by direct genotyping was 78.2% in CAD-REF and subsequent recalculation for rs139401390 missed a significant association with eGFR (p = 0.0991).

Since the discovery analysis suggested an association of rs139401390 with a mild reduction in kidney function in CAD patients, we assessed the odds ratio (OR) for eGFR < 60 ml/min/1.73 m^2^ in all LURIC patients with CAD (n = 2057, any eGFR) by rs139401390 genotype using imputed genomic data. The analysis revealed that the risk-allele combination (rs139401390_G/A) was associated with eGFR < 60 ml/min/1.73 m^2^ also in an adjusted (age, gender, BMI, diabetes, smoking, hypertension) analysis (adjusted p = 0.008, OR = 2.36, G/A + G/G vs A/A) (Table 3). Notably, the OR in the adjusted analysis for eGFR < 60 ml/min/1.73 m^2^ in the discovery sample including CAD patients with an eGFR between 30 and 75 ml/min/1.73 m^2^ was higher (adjusted p = 0.015, OR = 5.65, G/A + G/G vs A/A) (Table 3), while the OR for eGFR < 60 ml/min/1.73 m^2^ in the entire LURIC sample (CAD and no-CAD, any eGFR) was comparably low (adjusted p = 0.014, OR = 1.97, G/A + G/G vs A/A) (Table 3) and no effect was seen in patients without CAD (adjusted p = 0.571, OR = 1.36, G/A + G/G vs A/A) (Table 3).

**Table 3:** Risk of eGFR < 60 ml/min/1.73 m^2^ dependent on rs139401390.

rs139401390	n	eGFR < 60 ml/min/1.73 m^2^			
		Unadjusted		Adjusted*	
		OR (95% CI)	P	OR (95% CI)	P
**LURIC patients with CAD**					
A/A	1992	1		1	
G/A + G/G	65	1.90 (1.07–3.39)	0.029	2.39 (1.26–4.52)	0.008
**LURIC discovery sample (eGFR 30–75 ml/min/1**.**73 m**^**2**^ **AND CAD)**					
A/A	475	1		1	
G/A + G/G	14	4.87 (1.34–17.7)	0.016	5.64 (1.40–22.7)	0.015
**Total LURIC (CAD AND no-CAD)**					
A/A	2908	1		1	
G/A + G/G	97	1.76 (1.07–2.88)	0.025	1.97 (1.15–3.37)	0.013
**LURIC patients without CAD**					
A/A	916	1		1	
G/A + G/G	32	1.56 (0.59–4.14)	0.376	1.36 (0.48–3.90)	0.565

*Age, gender, Body Mass Index (BMI), diabetes, smoking, hypertension.

## Discussion

We report on a GWAS of eGFR in CAD patients of European ancestry with impaired kidney function. In the LURIC discovery sample, SNP rs139401390 located 58.8 kb upstream of *RNLS* was significantly associated with eGFR on the genome-wide level. Independent replication in patients of the CAD-REF study missed a significant association of the identified locus with eGFR. However, further analyses of rs139401390 associations within LURIC for patients with and without a comorbidity of impaired kidney function and CAD suggested a potential impact of rs139401390 on eGFR in this disease entity.

Our initial analysis suggested that rs139401390 located 58.8 kb upstream of *RNLS* was significantly associated with eGFR. Since association studies have demonstrated the affected genes are often located up to several megabases from the phenotype-associated variant, expression of the most proximal gene is not necessarily altered and rs139401390 might thus not mark *RNLS*. In addition, the *RNLS* locus has not yet been identified as a genetic risk locus for eGFR (or CKD) using genome-wide analyses. However, the coded protein renalase has frequently been discussed in CKD and CVD. Renalase was identified as a candidate involved in the regulation of cardiac function and blood pressure in 2005 by a cDNA library screen^19^. The initial study focused on the endocrine function of the kidney and reported on a novel flavin adenine dinucleotide–dependent amine oxidase termed renalase^19^. The data suggested that human renalase (the most highly expressed isoform being renalase (1) to be secreted into the blood by the kidney and identified significant renalase-depending breakdown of catecholamines *in vitro*, including dopamine, epinephrine and norepinephrine^19^. Addressing the link between the kidney and the cardiovascular system, subsequent studies used Sprague-Dawley rats injected with recombinant renalase, leading to a significant decrease in systolic, diastolic and mean arterial pressure as well as left-ventricular end-systolic and end-diastolic pressure, maximum left ventricular pressure, the rate of ventricular pressure change and heart rate^19^. In a follow-up study, it was reported that renalase knockout worsened kidney injury in animals^20^. It has also been proposed that renalase could modulate the intra-renal dopamine system, affecting sodium and phosphate excretion^21^.

The molecular mechanisms that mediate the acute activation of renalase *in vivo* are incompletely understood^22^. Renalase synthesis has been reported to be impaired in a renal artery stenosis rat model and the authors suggested that renal blood flow is a major determinant of renalase synthesis^23^. Notably, plasma renalase has been reported to be inactive while urinary renalase exerts amine oxidase activity under basal conditions^24^. Rapid activation of renalase by catecholamines within 30 sec has been observed^16^, which has led to the assumption that renalase may circulate as a proenzyme that requires specific signals for activation^22^. Recombinant renalase exerted protective effects in mouse models of acute kidney injury^25^ also independent of its ability to metabolize catecholamines^20^. Administration of renalase had also an intense and prolonged antihypertensive effect in an animal model of salt-sensitive hypertension^26^ and renalase perfusion exerted heart-protective effects on a cardiac ischemia mouse model^19,27^. More recently, and independent of its enzymatic properties, renalase has been suggested to exert cytokine functions that provide cell protection by activating a receptor‐mediated pro-survival signaling cascade^28^. While reduced renalase plasma levels have been reported in studies using animal models of CKD^24,29^, the correlation in humans is less definite. Initial studies suggested that CKD in humans is associated with renalase deficiency^19^ but recent reports including kidney^30^ and heart^31^ transplant patients as well as dialysis patients^32,33^ have led to controversial discussions^31,34,35^. Of note, discrepant findings have also been suggested to depend on the methods used to determine renalase levels^28,34^. Since the current study focused on the identification of disease-associated variants, we did not analyze if rs139401390 affects renalase levels.

While we detected a significant effect of rs139401390 genotypes on eGFR in the LURIC discovery cohort, no association was seen in the CAD-REF study. This observation might highlight an impact of rs139401390 risk alleles on eGFR in CAD patients with mildly reduced kidney function but not in CAD patients with moderately reduced kidney function and accompanying risk factors (age, hypertension, etc.). We investigated whether the identified rs139401390 genotype affected kidney function in the general population and conducted a separate association analysis of rs139401390 in 108,165 participants of the CKDGen consortium^12^, which consists of 20 predominantly population-based European studies. Notably, this analysis did not suggest an association of rs139401390 with eGFR in a population-based setting (data not shown), pointing to an important role for rs139401390 in CAD. Since separate analysis of rs139401390 risk alleles in the entire LURIC cohort revealed an elevated OR for eGFR < 60 ml/min/1.73 m^2^ in the presence of CAD, also dependent on the initial eGFR, rs139401390 might identify CAD patients that could benefit from more intensive clinical monitoring to prevent further reduction of kidney function.

Some limitations exist for the current analysis. Our study exclusively included participants of European ancestry and different results may be observed in other ancestral groups. We used an indirect measure of GFR, estimated by the MDRD equation, as direct determination of kidney function is not suitable on a population scale. The studies involved a one-point measurement of creatinine and the phenotypes of CKD initiation or progression have not been studied. No transformation procedure was used to adjust the eGFR distribution which could have affected the analysis. Observed low minor allele frequencies may have affected the presented findings and the calculation of the per-allele effect on eGFR. A major limitation is the missing replication of the suggested association of rs139401390 in an independent cohort, which may be based on study sample size and heterogeneity. Thus, future studies are warranted to validate our findings. While our discussion focuses on the potential impact of the identified variant on renalase expression, other genes might also be affected with a relevant impact on kidney function and we cannot exclude that the observed association was caused by other functional polymorphisms in genes other than *RNLS* in linkage disequilibrium with rs139401390. The transcription factor Gli2 has been reported to be an essential hedgehog signaling component involved in cardiogenesis^36–38^. The *SLC35C1-CRY2* locus on chromosome 11 may be involved in the development of hypertension^39^ and circadian gene expression involving cryptochrome-2 (CRY2) has been reported to be of importance in the vasculature and the heart^40^. Moreover, a synergistic effect of renalase and CKD on endothelin-1 in CAD patients has been suggested^41^.

In conclusion, we suggest that rs139401390 located to a region 58.8 kb upstream of *RNLS* may be associated with eGFR in CAD patients with a mild reduction of kidney function. Our study requires independent replication and may represent a potential basis for future studies on rs139401390, the enzyme renalase and the *RNLS* locus and their impact for the impairment of kidney function in CAD.
